# Deep Semantic-Preserving Reconstruction Hashing for Unsupervised Cross-Modal Retrieval

**DOI:** 10.3390/e22111266

**Published:** 2020-11-07

**Authors:** Shuli Cheng, Liejun Wang, Anyu Du

**Affiliations:** 1 College of Information Science and Engineering, Xinjiang University, Urumqi 830046, China; slcaydxju@stu.xju.edu.cn (S.C.); anydxju@xju.edu.cn (A.D.); 2 Key Laboratory of Signal Detection and Processing, Xinjiang Uygur Autonomous Region, Xinjiang University, Urumqi 830046, China

**Keywords:** cross-modal retrieval, semantic-preserving, spatial pooling, global covariance pooling

## Abstract

Deep hashing is the mainstream algorithm for large-scale cross-modal retrieval due to its high retrieval speed and low storage capacity, but the problem of reconstruction of modal semantic information is still very challenging. In order to further solve the problem of unsupervised cross-modal retrieval semantic reconstruction, we propose a novel deep semantic-preserving reconstruction hashing (DSPRH). The algorithm combines spatial and channel semantic information, and mines modal semantic information based on adaptive self-encoding and joint semantic reconstruction loss. The main contributions are as follows: (1) We introduce a new spatial pooling network module based on tensor regular-polymorphic decomposition theory to generate rank-1 tensor to capture high-order context semantics, which can assist the backbone network to capture important contextual modal semantic information. (2) Based on optimization perspective, we use global covariance pooling to capture channel semantic information and accelerate network convergence. In feature reconstruction layer, we use two bottlenecks auto-encoding to achieve visual-text modal interaction. (3) In metric learning, we design a new loss function to optimize model parameters, which can preserve the correlation between image modalities and text modalities. The DSPRH algorithm is tested on MIRFlickr-25K and NUS-WIDE. The experimental results show that DSPRH has achieved better performance on retrieval tasks.

## 1. Introduction

With the growth of multimodal data on the web, cross-modal retrieval has become an interactive method in visual and language understanding. Because of the high retrieval speed and low storage space, hashing algorithm has become a real-time processing method for multi-modal complex scenes to give machines cognitive and understanding capabilities. This is a method that can map high-dimensional data to low-dimensional space and guarantee the similarity of original high-dimensional data in low-dimensional space. Hash algorithms include single-modal hash algorithms [1,2,3,4] and cross-modal hash algorithms [5,6]. Compared with the single-mode hash algorithm, cross-modal retrieval focuses on the mutual retrieval tasks between different modalities.

**Figure 1:**
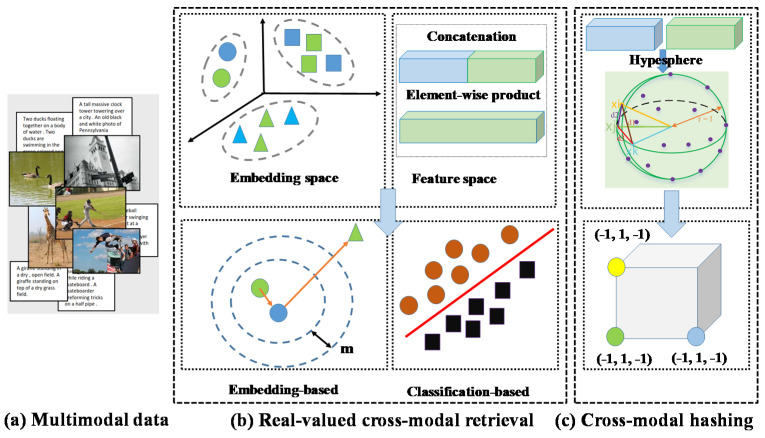
Overview of cross-modal retrieval. (**a**) shows multi-modal data, (**b**) shows real-valued cross-modal retrieval and (**c**) is cross-modal hash retrieval. (**b**) mainly explores the matching process between image regions and text words, while (**c**) mainly maps images and text in the same Hamming space to measure the similarity of image and text modalities. The cross-modal hash retrieval algorithm is a binary representation, so it fits the computer calculation method, so the cross-modal hash has less storage and retrieval speed while mining modal information.

Cross-modal retrieval tasks include real-value cross-modal retrieval and cross-modal hashing retrieval. Real-valued cross-modal retrieval processing methods include classification-based and embedded-based, and its typical representative works include stacked cross attention network (SCAN) [7] and saliency-guided attention network (SAN) [8]. In the two modalities of image and text, image-text retrieval is mainly to realize the matching problem of image and text to explore the correspondence between image regions and sentence words. In order to further capture the modal semantic information, reduce the amount of calculation and accelerate the retrieval speed, the deep hash algorithm has become the mainstream method of image-text retrieval to measure modal semantic information in the Hamming space [9,10,11]. Figure 1 shows the overall architecture of cross-modal retrieval.

Although the cross-modal hash algorithm can capture modal semantics quickly and efficiently, modal semantic alignment and semantic reconstruction still face great challenges. In unsupervised cross-modal hashing, Su et al. proposed [12] a semantic reconstruction framework to mine modal semantic information. Considering semantic alignment, Zhang et al. proposed [13] a deep semantic alignment hashing algorithm to explore the inner connection of modal semantics. In terms of the network model, Hu proposed [14] unsupervised knowledge distillation to create something from nothing. Bai et al. proposed [15] to resist loss to associate different modal semantics. Gu et al. proposed [16] an adversary-guided attention module to enhance feature learning. Shen et al. proposed [17] automatic twin-bottleneck hashing, whose main goal is to generate similarity graphs based on graph convolutional network (GCN). These studies have improved the performance of cross-modal hash retrieval to a certain extent. In current research, researchers have found that second-order statistics can speed up network convergence compared to stochastic gradient descent (SGD) [18], but these studies are still in the enlightenment stage, and these studies have not yet been explored in cross-modal hashing. In addition, modal semantic reconstruction is still the difficulty of cross-modal hashing.

Encouraged by unsupervised cross-modal hashing algorithms [12,13,14,15,16,17], global covariance pooling [18,19,20] and spatial pooling network module [21], we propose a novel deep semantic-preserving reconstruction hashing (DSPRH). The algorithm combines spatial and channel semantic information, and mines modal associated semantic information based on adaptive self-encoding and joint semantic reconstruction loss. The main contributions are summarized as follows: (1) We introduce a new spatial pooling network module based on tensor regular-polymorphic decomposition theory to generate rank-1 tensor to capture high-order context semantics, which can assist the backbone network to capture important contextual modal semantic information. (2) Based on optimization perspective, we use global covariance pooling to capture channel semantic information and accelerate network convergence. In feature reconstruction layer, we use two bottlenecks auto-encoding to achieve visual-text modal interaction. (3) In metric learning, we design a new semantic-preserving reconstruction loss (using semantic alignment and cosine triplet loss function) to optimize model parameters, which can preserve the correlation between image modalities and text modalities. (4) The DSPRH algorithm is tested on two datasets (MIRFlickr-25K and NUS-WIDE). The experimental results show that DSPRH has achieved better performance on image2text and text2image retrieval tasks.

The remaining chapters of this paper are organized as follows: Section 2 is related work. Section 3 describes the proposed deep semantic-preserving reconstruction hashing method. Section 4 presents the experimental results and analysis. Section 5 summarizes the work of this paper and proposes future research plans.

## 2. Related Work

Image-text retrieval is the technical support for building a new generation of cross-media search system and cross-media monitoring system, and it is the core technology of cross-media analysis and reasoning, intelligent cognition and understanding. Cross-modal retrieval has become a universal search engine and entertainment tool, which facilitates people’s production and life. For example, Taobao shopping, Jingdong shopping, and Kuaishou. These can realize the mutual search of images and texts. In the massive multi-modal heterogeneous data, how to quickly and accurately find different modal data has become a research hotspot and difficulty. The goal of cross-modal hash retrieval is to use less storage space to quickly retrieve modal information. Image-text retrieval is an important means of current question answering systems and video image description, and it is also an important field of machine understanding. In this section, we introduce deep cross-modal hashing and key modular knowledge.

### 2.1. Cross-Modal Hashing

With the rapid increase of network data, deep cross-modal retrieval has become a research hotspot in recent years. Many researchers have carried out a lot of research on loss function design and network model construction. Specifically, Li et al. proposed self-supervised adversarial hashing [5] to discover high-level semantic information. Wu et al. proposed unsupervised deep cross-modal hashing [6] to integrate deep learning and matrix factorization. Zhang et al. proposed unsupervised generative adversarial cross-modal hashing [9] to perform unsupervised representation learning. Liu et al. proposed matrix tri-factorization hashing [10] to explore effective objective functions. Wang et al. proposed semantic-rebased cross-modal hashing [11] to achieve unsupervised learning. From the perspective of semantic reconstruction and alignment, joint semantic reconstruction and semantic alignment have received extensive attention in retrieval [12,13,14,15,16]. Hu et al. proposed collective reconstructive embeddings [22] to deal with heterogeneous challenges. Chen et al. proposed shallow modal representation [23]. Some research work [24,25] has carried out research on cross-modal retrieval network models, the main goal of which is to obtain robust modal semantic description. There is also some research work [26,27,28,29,30] to improve the loss function to maintain semantic relevance. Nie et al. proposed [31] multi-scale fusion transmembrane retrieval to effectively use modal information. Lin et al. proposed [32] mask cross-modal hashing to capture inter-modal semantic information. Zhang et al. proposed [33] a multi-pathway unsupervised hashing method based on a generative adversarial network (GAN) to utilize multi-modal information. In addition, Zhang et al. proposed [34] semi-supervised GAN hashing to mine image modal and text modal information. Although cross-modal retrieval faces great challenges, these researches have greatly promoted the progress of cross-modal hashing technology.

### 2.2. Second-Order Covariance Pooling

In recent years, researchers have found that second-order optimization has better network optimization performance than SGD. Li et al. improved [20] the performance of large-scale visual recognition tasks based on matrix power normalization. The iSQRT-COV is proposed [19] to accelerate network convergence. In order to deal with three-dimensional tensors, the researcher proposed GSoP-Net block [35], which is a network module that is easy to embed. Wang et al. discussed [18] the benefits of global covariance pooling (GCP) to the network from loss optimization and gradient prediction. The research shows that second-order statistical optimization can enhance the feature expression ability of convolutional neural networks. Specifically, when the backbone network is fixed, compared with global average pooling, global covariance pooling has better feature description capabilities. In addition, Wang et al. gave [36] a better feature representation method based on GCP. Research has found that second-order optimization algorithms (such as GCP) can accelerate network convergence algorithms and improve overall network performance compared to first-order optimization (such as global mean pooling (GAP)).

In summary, loss function and model optimization are currently the main techniques for cross-modal retrieval. Encouraged by DJSRH [12] and DSAH [13], our main goal is to explore a new metric learning method to complete cross-modal retrieval tasks. Specifically, the core idea of DJSRH [12] is to reconstruct a joint semantic loss to associate two modal information (image feature space and text feature space). This technology was published in ICCV 2019. Compared with DJSRH [12], the DSAH [13] algorithm increases the semantic alignment loss based on the joint semantic reconstruction loss. DSAH [13] comprehensively utilizes feature space semantics and Hamming space semantic information, so its retrieval performance is better than DJSRH. DSAH [13] was proposed in ICMR 2020. Encouraged by the DJSRH [12] and DSAH [13] algorithms, we propose the DSPRH algorithm. We use the idea of feature fusion to reconstruct the image feature space, and propose a new cross-modal retrieval loss function based on considering multiple modal semantic alignment loss, pairwise loss and cosine triple loss. 

## 3. Our Method

In the current research, researchers are focusing on accelerating network convergence and cross-modal hash information reconstruction. The goal of these works is to enhance the ability of feature expression to explore visual-language understanding tasks. Different from the current cross-modal hash research, our main contributions are as follows: (1) We introduce a new spatial pooling to assist the backbone network AlexNet to capture important contextual semantic information. (2) In terms of network training and feature extraction, we use GCP to accelerate network convergence and use GCP to capture channel semantic information. In addition, we use two bottlenecks auto-encoding to achieve visual-text modal interaction. (3) In metric learning, we propose a new semantic-preserving reconstruction loss, which can reserve important modal semantic information.

### 3.1. The Overall Architecture of The Algorithm

**Figure 2:**
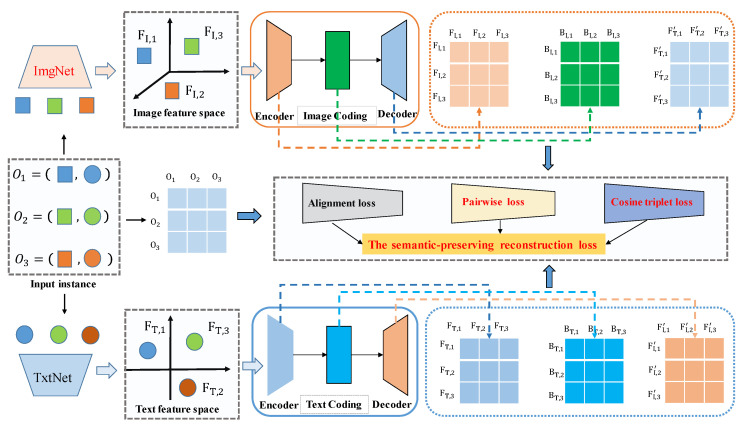
The overall architecture of the deep semantic-preserving reconstruction hashing.

Figure 2 shows the architecture of DSPRH. Specifically, Figure 2 contains three main steps. DSPRH uses ImgNet to extract image feature space, and uses TxtNet to extract text feature space. Where ImgNet uses GCP-based optimization to accelerate network convergence. In addition, ImgNet’s unique technology is based on SPNet to extract important modal spatial features, and based on GCP to obtain important modal channel features. In order to further reconstruct the modal semantic information, we used the two bottlenecks module to reconstruct the text feature space and the image feature space, which is a self-encoding process. Finally, we proposed a new semantic-preserving reconstruction loss. 

This work is different from the existing unsupervised cross-modal hashing work. Specifically, we introduced a new reconstruction feature space and used alignment loss to reconstruct modal information. In addition, we used pairwise loss and cosine triple loss to preserve the semantic information of image modal and text modal. The experiment showed the effectiveness of semantic-preserving reconstruction loss. 

In Figure 2, the paired semantic information entered the text network to obtain the text feature space, and the paired semantic information entered the image network to obtain the image feature space. Visual-language feature extraction consists of image feature space and text feature space. Visual-language feature extraction consists of image feature space and text feature space. Visual-language feature extraction is shown in Section 3.2. 

**Figure 3:**
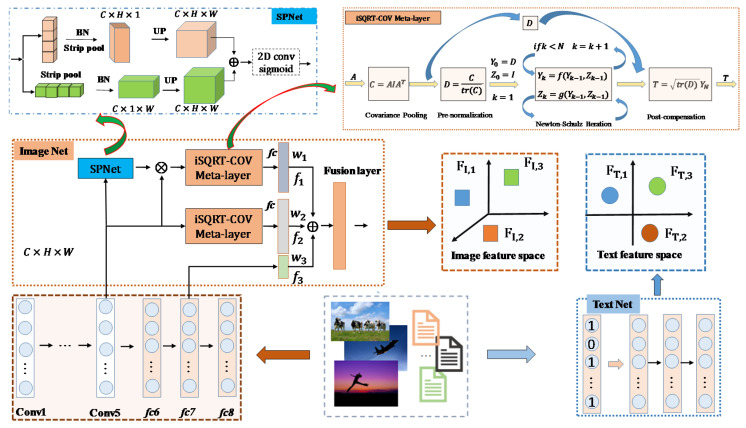
Image-text feature extraction process. In Figure 3, the backbone network of image features is the pre-trained AlexNet, which is the current mainstream cross-modal hash architecture to ensure comparison fairness.

In Section 3.2, we mainly introduce visual-language feature extraction. We propose a feature fusion strategy, which includes 3 types of features. The fully connected layer did not need to go through the iSQRT-COV layer to obtain the feature description of the image, and the output of the convolutional layer needed to go through the iSQRT-COV layer mapping to obtain the high-level semantics of the image. The iSQRT-COV was proposed [19] to accelerate network convergence, and iSQRT-COV can work with convolutional networks to obtain high-level image semantic description, and its output form was consistent with the output form of the fully connected layer. The full name of the iSQRT-COV module is iSQRT-COV meta-layer. The three types of feature outputs were weighted and fused, which we named the fusion layer. In this way, we reconstruct the image feature space. The image-text feature extraction process is shown in Figure 3. In Figure 3, SPNet block [21] is a low-rank expression convolution kernel, which is a strip pooling operation to reconstruct the semantic feature map, which was proposed in the CVPR 2020 conference.

The DSPRH mainly includes three main technologies: (1) ImgNet’s backbone network is AlexNet, and we used spatial pooling and GCP optimization to extract robust image descriptions. (2) After ImgNet and TxtNet extract visual and language features, respectively, the two bottleneck coding model was used to reconstruct text features and image features. (3) We proposed a new loss function, the full name is semantic-preserving reconstruction loss. This loss function includes alignment loss, pairwise loss and cosine triple loss. Research work shows that this modal semantic reconstruction can significantly improve the performance of unsupervised cross-modal hashing.

### 3.2. Visual-Language Feature Space

Visual-language feature extraction is one of the key elements of DSPRH in Figure 2. We detailed the visual-text feature extraction steps in Figure 3. For fair comparison, we used AlexNet as the backbone network for visual feature extraction. Visual feature extraction consists of three parts. Based on the pre-trained AlexNet model, we extracted the *fc7* feature *f3*. We used the feature of *conv5* as iSQRT-COV meta-layer [19] to capture the semantic information between channels, and the feature was *f2* after passing through the fully connected layer. In order to quickly capture the spatial modal information, we input the features of *conv5* into the SPNet block [21], and then cascaded the iSQRT-COV meta-layer [18,19,35,36] and the fully connected layer fc to extract the image feature *f1*. Where the weights of *f1*, *f2*, and *f3* were *w1*, *w2* and *w3* in the image feature fusion layer. The text feature space was constructed based on Text Net. In order to maintain the fairness of text feature extraction, we kept the Text Net model parameter settings consistent with most existing works [11,12,13,15,16].

### 3.3. Feature Reconstruction Layer

In Figure 2, the dimension of the image feature space and the dimension of the text feature space were inconsistent, and the dimension of the hash code was much smaller than the dimension of the image and text feature space. In order to align image-text semantics, inspired by twin-bottleneck hashing [17], we introduced an image feature reconstruction layer after the image feature space, and we introduced a text feature reconstruction layer after the text feature space. In Figure 2, we introduced the feature reconstruction layer, which included image feature reconstruction layer and text feature reconstruction layer. In the image feature reconstruction layer, it included image encoder, image coding and image decoder. In the text feature reconstruction layer, it included text encoder, text coding and text decoder. 

In Figure 2, through the feature reconstruction layer, we aligned image and text feature semantics, and aligned image and text hash code semantics. Therefore, we proposed alignment loss. Considering that there were image hash code output and text hash code output in cross-module hashing, we proposed pairwise loss to optimize the DSPRH model parameters. Similar to the idea of pairwise loss, inspired by the triple loss, we proposed the cosine triple loss to further optimize the network parameters. Based on the above considerations, we proposed semantic-preserving reconstruction loss, which is a new metric learning method that considers both semantic alignment and modal semantic association.

The definition of basic symbols is shown in Table 1.

Assuming we have M semantic pairs, we can define it as:

(1) $$O={\left\{O_{k}\right\}}_{i=1}^{M}={\left\{\left\{I_{k},T_{k}\right\}\right\}}_{i=1}^{M}$$

where the feature of $I_{k}$ is $F_{I}\epsilon {\mathbb{R}}^{M\times D_{I}}$ and the feature of $T_{k}$ is $F_{T}\epsilon {\mathbb{R}}^{M\times D_{T}}$. *i* represents the semantics of the *i*-th image.

We defined the encoder function, coding function and decoder function as *Enc*, *Cod* and *Dec,* respectively. In image feature reconstruction layer, the basic definition is as follows:

(2) $$H_{I}=Enc\left(F_{I};\theta_{I}\right)$$

(3) $$B_{I}=Cod\left(H_{I}\right)$$

(4) $$F_{T}^{\prime }=Dec\left(B_{I};\eta_{I}\right)$$

where $H_{I}$ is the output of the image coding layer, $B_{I}$ is the image hash code and $F_{T}^{\prime }$ is the reconstructed text feature space. $\theta_{I}$ and $\eta_{I}$ are encoder layer and decoder layer parameters, respectively. 

In the same way, we can get text feature encoding $H_{T}$, text hash code $B_{T}$ and reconstructed image feature space $F_{I}^{\prime }$.

(5) $$H_{T}=Enc\left(F_{T};\theta_{T}\right)$$

(6) $$B_{T}=Cod\left(H_{T}\right)$$

(7) $$F_{I}^{\prime }=Dec\left(B_{T};\eta_{T}\right)$$

where $\theta_{T}$ and $\eta_{T}$ are text encoder layer and text decoder layer parameters, respectively.

### 3.4. Semantic-Preserving Reconstruction Loss

This loss includes alignment loss (reconstruction loss), pairwise loss and cosine triple loss.

#### 3.4.1. The Entire Alignment Loss

The reconstruction loss $L_{F}$ is defined as follows:

(8) $$L_{F}=\sum ∥F_{I}^{\prime }-F_{T}{∥}^{2}+\sum ∥F_{T}^{\prime }-F_{I}{∥}^{2}$$

Based on the diagonalization trend of the similarity matrix program between modalities, the ranking loss is proposed to effectively associate the modal semantic information.

(9) $$L_{R1}=\sum_{i=1}^{M}∥1-S_{I,T}^{B}{\left(i,i\right)∥}^{2}$$

(10) $$L_{R2}=\frac{1}{2}\sum_{i=1}^{M}\sum_{j=1}^{M}∥S_{I,T}^{B}\left(i,j\right)-S_{I,T}^{B}{\left(j,i\right)∥}^{2}$$

(11) $$L_{R}=L_{R1}+L_{R2}$$

where $L_{R1}$ represents the semantic alignment loss within the modal, and $L_{R2}$ represents the semantic alignment loss between the modalities. $S_{I,T}^{B}$ is the similarity matrix based on B, where *I* is the image modality and *T* is the text modality. $L_{R}$ is the sort alignment loss.

In order to further correlate the modal semantics, the fusion feature matrix is defined as follows:

(12) $$S_{I,T}^{F}=\lambda S_{I,I}^{F}+\left(1-\lambda \right)S_{T,T}^{F}$$

where λ is a hyperparameter. $S_{I,I}^{F}$ is the spatial similarity of image semantic features and $S_{T,T}^{F}$ is the spatial similarity of image semantic features.

The semantic alignment loss of feature space and hash code space is $L_{w}$ to reduce the semantic gap.

(13) $$L_{W}=\sum_{i=1}^{M}∥kS_{X,X}^{F}-S_{I,T}^{B}{∥}^{2}+\mu \sum_{i=1}^{M}∥kS_{I,T}^{F}-S_{X,Y}^{B}{∥}^{2}$$

where (X,X)ϵ{(I,I),(T,T)}, (X,Y)ϵ{(I,I),(T,T),(I,T)}. $S_{X,Y}^{B}$ is the similarity matrix based on B. $S_{X,X}^{F}$ is the similarity matrix based on F in (X,X). *u* is the hyperparameter to balance the importance of different modalities.

The entire alignment loss ($L_{1}$) of DSPRH is defined as follows:

(14) $$L_{1}=L_{F}+L_{R}+L_{W}$$

#### 3.4.2. Cosine Triplet Loss

Cosine triple is a loss function in which triples and cosines are nested. The image hash code is $x_{i}$, $y_{j}^{+}$ is a text hash code related to $x_{i}$, and $y_{k}^{-}$ is text hash code not related to $x_{i}$. 

The relevant definition of the loss function of the cosine triplet is as follows:

(15) $$L_{I\to T}=\sum_{i,j,k}\max\left(\cos\left(x_{i},y_{k}^{-}\right)-\cos\left(x_{i},y_{j}^{+}\right)+\nu ,0\right)$$

where $L_{I\to T}$ is the cosine triple loss of the image search text. Cos(x,y) represents the cosine distance metric between x and y.

(16) $$L_{T\to I}=\sum_{i,j,k}\max\left(\cos\left(y_{i},x_{k}^{-}\right)-\cos\left(y_{i},x_{j}^{+}\right)+\nu ,0\right)$$

where $L_{T\to I}$ is the cosine triple loss of the text search image. Text hash code is $y_{i}$, $x_{j}^{+}$ is a image hash code related to $y_{i}$, and $x_{k}^{-}$ is image hash code not related to $y_{i}$.

The entire cosine triple loss ($L_{2}$) of DSPRH is defined as follows:

(17) $$L_{2}=L_{I\to T}+L_{T\to I}$$

#### 3.4.3. Pairwise Loss

We define the pairwise loss of DSPRH, and its goal is to reserve modal semantic information. In the semantic pair, the image hash code is $d_{i}$, the text hash code is $d_{j}$ and the semantic similarity matrix S. $s_{ij}$ is the similarity between *i*-th image and *j*-th text. Based on the maximum likelihood criterion, we can define the cross-modal pairwise loss of DSPRH. Its definition is as follows:

(18) $$L_{3}=-logP\left(S|B\right)=\sum_{s_{ij}\epsilon S}\left(s_{ij}\Omega_{ij}-\left(1+exp^{\Omega_{ij}}\right)\right)$$

where $L_{3}$ is the cross-modal pairwise loss, $\Omega_{ij}=\frac{1}{2}d_{i}^{T}d_{j}$.

#### 3.4.4. Semantic-Preserving Reconstruction Loss (All Loss of DSPRH)

Based on alignment loss, pairwise loss, and cosine triple loss, the total loss of DSPRH is defined as follows:

(19) $$L_{All}=\alpha L_{1}+\beta L_{2}+\gamma L_{3}$$

where α, β and γ are three hyperparameters, which mainly reflect the importance of the three losses to network optimization. $L_{1}$ is the alignment loss and reconstruction loss of DSPRH, $L_{2}$ is the cosine triple loss of DSPRH and $L_{3}$ is the cross-modal pairwise loss of DSPRH.

## 4. Experiments

In this section, we test the performance of DSPRH on two public datasets (MIRFlickr-25K and NUS-WIDE). We compare the performance of the DSPRH method with some state-of-the-art methods.

### 4.1. Datasets, Evaluation Indicators and Baselines

Datasets introduction: We evaluated the DSPRH algorithm on two datasets; MIRFlickr-25K and NUS-WIDE. Specifically, MIRFlickr-25K contains 25,000 image-text semantic pairs. Each semantics is labeled with one of 24 categories. In the experimental part, we used 20015 semantic pairs in our experiment, these settings are based on the current cross-modal hash retrieval algorithm settings [12,13,14,15,16]. Among them, 2000 semantic pairs were used for query.

Another dataset was NUS-WIDE. The data set contains 269,498 semantic pairs and defines 81 categories. During the experiment, 186,577 semantic pairs and the top 10 most frequent annotations were executed in our experiment. This experiment was also performed according to the current standards of most cross-modal hashing algorithms [12,13,14,15,16]. Among them, 2000 semantic pairs were used for query.

Experimental details: For a fair comparison, our backbone network selects the AlexNet network, which is also the current cross-modal hashing general setting [6,12,13,29]. The AlexNet used by DSPRH is a pre-trained model used in the Image network. We added ResNet34 as the backbone network mainly to illustrate the scalability of our algorithm, which was just an additional experiment of the DSPRH algorithm. Performing related experiments on MIRFlickr-25K, we extracted 4096-dimensional image features and extracted 1386-dimensional bag-of-words (BOW) features for each text. When performing experiments on the NUS-WIDE dataset, we extracted 4096-dimensional features of each image and 500-dimensional BOW features of each text. λ is a hyperparameter in formula 12, where λ is 0.9 based on pre-work [12]. In formulas 15 and 16, the hyper-parameter ν is equal to 0.001. In Equation (19), the weight distribution of the three loss functions (α, β and γ) is described in the section of ablation analysis.

Evaluation indicators: The mean average precision (MAP) is a general index to measure the performance of cross-modal retrieval algorithms, which can reflect the average of the average retrieval accuracy of all query samples. In this article, cross-modal retrieval involved 6 evaluation indicators, including Image2text MAP@50, Image2text MAP, Text2image MAP@50, Text2image MAP, MAP@50 and MAP.

Baselines: This paper selected 8 algorithms to perform MAP comparison, and 17 algorithms to perform MAP@50 comparison to evaluate algorithm performance. To test the effectiveness of the DSPRH algorithm, we repeated the DJSRH [12] and DSAH [13], because the DSPRH enlightenment idea originated from DJSRH [12] and DSAH [13]. Specifically, these baselines are described as follows: UKD [14] (CVPR 2020), SRCH [11] (IJCAI 2020), MGAH [33] (TMM 2020), UGACH [9] (AAAI 2018), DJSRH [12] (ICCV 2019), DSAH [13] (ICMR 2020), DBRC [28] (TMM 2019), CRB [22] (TIP 2019), DADH [15] (ICMR 2020), AGAH [16] (ICMR 2019), UDCMH [6] (IJCAI, 2018), SCH-GAN [34] (TOC, 2020); DSPOH [29] (TNNLS, 2019), SCRATCH [23] (TCSVT, 2019), MTFH [10] (TPAMI, 2019), EGDH [37] (IJCAI, 2019), DMFH [31] (TCSVT, 2020), BATCH [26] (TKDE, 2020), ATFH-N [25] (TETCI, 2020), MDCH [32] (TMM, 2020), CPAH [27] (TIP, 2020), MLCAH [30] (TMM, 2020) a d SSAH [5] (CVPR, 2018).

### 4.2. DSPRH and Baselines Comparison

#### 4.2.1. Performance Comparison on MIRFlickr-25K

Most current algorithms actually focus on MAP@50 and MAP when calculating the performance of cross-modal hashing algorithms. MAP is the result of all query semantics, and MAP@50 mainly calculates MAP for the first 50 elements of the query. Table 2 shows the performance comparison on MIRFlickr-25K. In Table 2, inspired by the two algorithms corresponding to the blue font [12,13], the alignment loss of DSPRH partly comes from these two algorithms, so we repeated these two algorithms [12,13] and calculated the corresponding MAP and MAP@50 results. In addition, we analyzed the work of cross-modal hashing algorithms in recent years. We found that we could mine modal semantic information from the aspects of semantic spatial information reconstruction and loss function, which can greatly improve performance.

Compared with some current frontier works (based on graph convolution, model pruning and GAN), our proposed DSPRH has better performance. Compared with these two algorithms (DSAH [13] and DJSRH [12]), DSPRH is based on the tensor generation module (TRM), which generates many level 1 tensors to capture contextual feature fragments. Then, we used these level 1 tensors to recover high-order context features through the TRM. Therefore, our proposed DSPRH can obtain the semantic modal information of the fusion context on the one hand with less computational complexity. In addition, the DSPRH algorithm is based on three loss functions to optimize network parameters, confirming the effectiveness of DSPRH.

Specifically, if we choose AlexNet as the backbone network, for the image2text retrieval performance, DSPRH compared to DSAH [13] MAP@50 improved by 4.1%, 4.9%, 3.4% and 3.2% at 16, 32, 64 and 128 bits, respectively. For text2image retrieval performance, DSPRH MAP@50 has been improved by 3.3%, 2.5%, 3.1% and 3.5% at 16, 32, 64 and 128 bits, respectively, compared to DSAH [13]. Compared to baselines, both image2text MAP and text2image MAP, DSPRH showed better performance. The ResNet34 as the backbone network is to verify the scalability of DSPRH, which can further significantly improve the performance of DSPRH.

#### 4.2.2. Performance Comparison on NUS-WIDE

Table 3 shows performance comparison on NUS-WIDE. Similar to Table 2, we compare the performance of MAP and MAP@50, respectively. Encouraged by DJSRH [12] and DSAH [13], we proposed DSPRH. The blue font corresponds to the main comparison algorithm, and the red part is the proposed algorithm (including AlexNet for the backbone network and ResNet34 for the backbone network). AlexNet as the backbone network was mainly to evaluate the effectiveness of the algorithm, which was a fair comparison experiment. ResNet34 can further improve the performance of the algorithm, which was used to illustrate the network scalability of the algorithm in this paper.

Specifically, DSAH [13] and DJSRH [12] were the two most relevant baselines in this article. Because the performance of DSAH [13] was better than DJSRH [12], we mainly analyzed the performance comparison between DSPRH and DSAH [13]. If we choose AlexNet as the backbone network, for the image2text retrieval performance, DSPRH compared to DSAH [13] MAP@50 improved by 2.5%, 3.6%, 4.2% and 3.5% at 16, 32, 64 and 128 bits, respectively. For text2image retrieval performance, DSPRH MAP@50 has been improved by 1.4%, −0.1%, 2.8% and 1.4% at 16, 32, 64 and 128 bits, respectively, compared to DSAH [13].

Based on MAP to measure cross-modal hashing retrieval performance, we also select 8 baselines for evaluation. If we choose AlexNet as the backbone network, for the image2text retrieval performance, DSPRH MAP compared to DSAH [13] improved by 6.9%, 8.4%, 8.2% and 9.5% at 16, 32, 64 and 128 bits, respectively. For text2image retrieval performance, DSPRH MAP improved by 8.5%, 8.9%, 10.5% and 6.7% at 16, 32, 64 and 128 bits, respectively, compared to DSAH [13]. DSPRH can be further expanded. When we choose ResNet34 as the backbone network, the performance of DSPRH algorithm can be further improved. In short, DSPRH achieved better performance compared to baselines. DSPRH was tested on two public data sets. Experimental results show that our algorithm has better retrieval performance and can fully mine modal context semantic information.

### 4.3. Ablation Analysis

#### 4.3.1. Loss Function Ablation Analysis

In this section, we analyzed the influencing factors of DSPRH, including loss function influence, modal semantic information fusion influence and hyperparameter sensitivity analysis. The loss function of DSPRH is semantic-preserving reconstruction loss (full loss). Full loss includes alignment loss, cosine triple loss and pairwise loss as shown in Figure 2 and Equation (19). Therefore, we performed ablation analysis on the loss function part. We show the loss function ablation analysis in Table 4, which represents the loss function ablation analysis under the hash code length of 128, 64, 32 and 16. The figures of the loss function ablation analysis is described in Appendix A.

**Figure A1:**
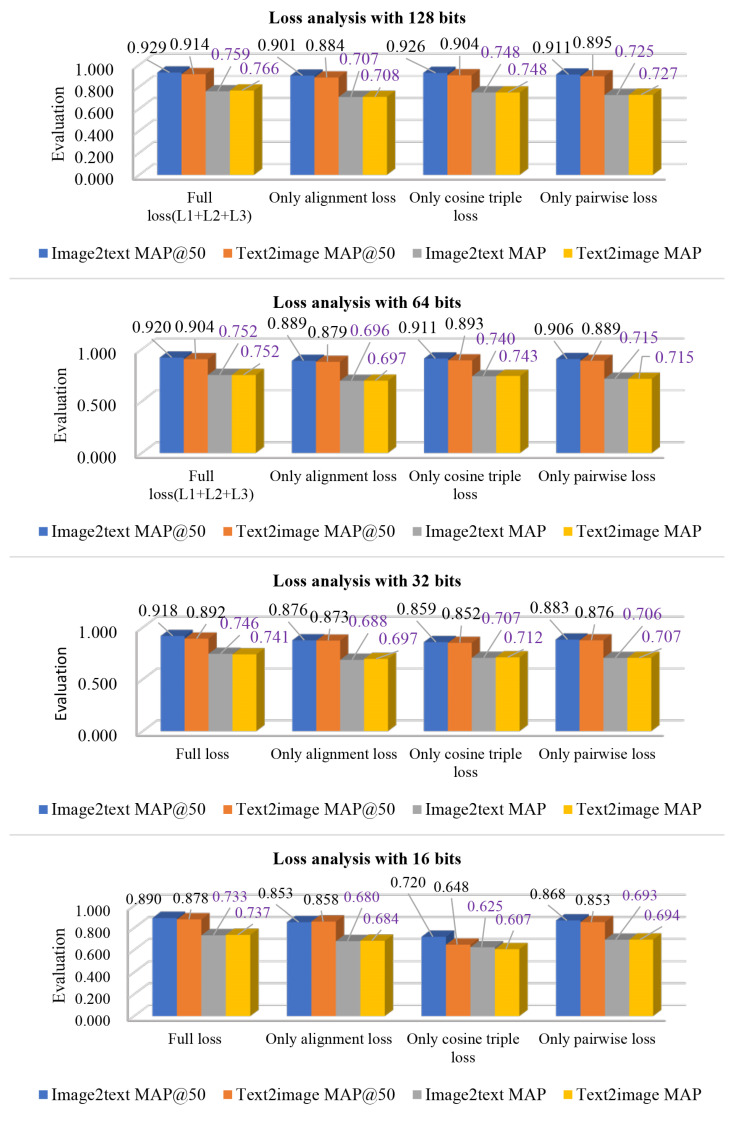
Loss function ablation analysis with different bits on MIRFlickr-25K.

Loss ablation analysis on MIRFlickr-25K: We discuss the loss function ablation analysis with different bits in Figure A1. In Figure A1, we found the best performance of our proposed semantic-preserving reconstruction loss (full loss). The cosine triple loss ($L_{2}$) contributed the most to full loss compared to alignment loss and pairwise loss with 128 bits. In Figure A1, we found the best performance of our proposed semantic-preserving reconstruction loss (full loss) with 64 bits. The cosine triple loss ($L_{2}$) contributed the most to full loss compared to alignment loss and pairwise loss with 64 bits. In Figure A1, we found the best performance of full loss with 32 bits. The pairwise loss ($L_{3}$) contributed the most to full loss compared to alignment loss and cosine triple loss with 32 bits. In Figure A1, we found the best performance of full loss with 16 bits. The pairwise loss ($L_{3}$) contributed the most to full loss compared to alignment loss and cosine triple loss with 16 bits.

In short, we found that DSPRH can get the best performance on MIRFlickr-25K in full loss (alignment loss, cosine triple loss and pairwise loss). When hash bits were 64 bits and 128 bits, the cosine triple loss will benefit the most from full loss. When hash bits were 32 bits and 16 bits, the pairwise loss will benefit the most from full loss.

**Figure A2:**
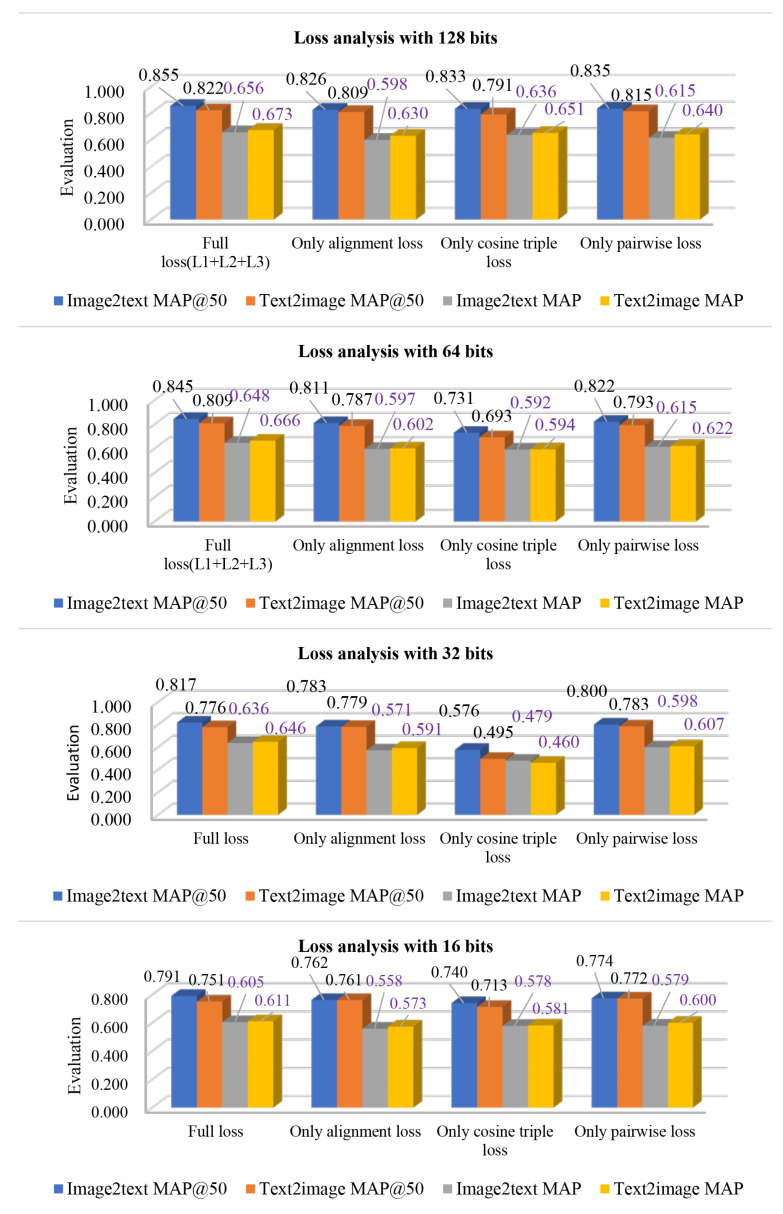
Loss function ablation analysis with different bits on NUS-WIDE.

Loss ablation analysis on NUS-WIDE: We discuss the loss function ablation analysis with different bits in Figure A2. In Figure A2, we found the best performance of our proposed semantic-preserving reconstruction loss (full loss). In Figure A2, pairwise loss contributed the most to our proposed semantic-preserving reconstruction loss with different bits. The order of the advantage of loss function was: Pairwise loss > alignment loss > cosine triple loss.

Loss ablation analysis summary: Table 4, Figure A1 and Figure A2 show the loss function ablation analysis of DSPRH under different datasets. The experimental results showed that pairwise loss achieves good results on both data sets. This loss was mainly used in image single-mode retrieval research and was less concerned by cross-mode retrieval. However, in cross-modal retrieval, the meaning of semantic information interaction between image modality and text modality was consistent with the meaning of single-modal image for information interaction, so this loss can also be applied to cross-modal retrieval tasks.

Specifically, the performance of cosine triplet loss was better than pairwise loss at a higher number of bits (64 bits and 128 bits), and the performance of pairwise loss was better than alignment loss in Figure A1. The order of the advantage of loss function on MIRFlickr-25K was: Full loss > cosine triple loss > alignment loss > pairwise loss. On the contrary, the performance of the pairwise loss was better than the alignment loss at a lower number of bits (32 bits and 16 bits), and the performance of the alignment loss was better than the cosine triplet loss in Figure A1. The order of the advantage of loss function on MIRFlickr-25K was: Full loss > pairwise loss > alignment loss > cosine triple loss. In the NUS-WIDE dataset with a large amount of data, pairwise loss contributed the most to our proposed semantic-preserving reconstruction loss with different bits. The order of the advantage of loss function was: Full loss > pairwise loss> alignment loss> cosine triple loss. Therefore, the loss function proposed in this paper had better performance and achieved better retrieval performance than current cross-modal retrieval algorithms.

#### 4.3.2. Feature Space Fusion Analysis

In Figure 3, visual feature extraction included three types: $f_{1}$, $f_{2}$ and $f_{3}$. The weights of the three features were $w_{1}$, $w_{2}$ and $w_{3}$. $f_{3}$ was a fully connected feature of AlexNet. $f_{2}$ was a feature of AlexNet’s conv5, GCP and fully connected layer cascade. $f_{1}$ was a feature of strip pool block, GCP and fully connected cascade. In Figure 3, $w_{1}$ + $w_{2}$ + $w_{3}$ = 1, in order to simplify the parameter, we assume $w_{2}$ was equal to $w_{1}$. We also carried out relevant experiments to verify, and the results found that when $w_{2}$ was equal to $w_{1}$, the retrieval performance was the best. Next we showed the relevant ablation analysis. Overall, $w_{1}$, $w_{2}$ and $w_{3}$ were 0.25, 0.25 and 0.5 on MIRFlickr-25K, respectively. $w_{1}$, $w_{2}$ and $w_{3}$ were 0.125, 0.125 and 0.75 on NUS-WIDE, respectively.

**Figure 4:**
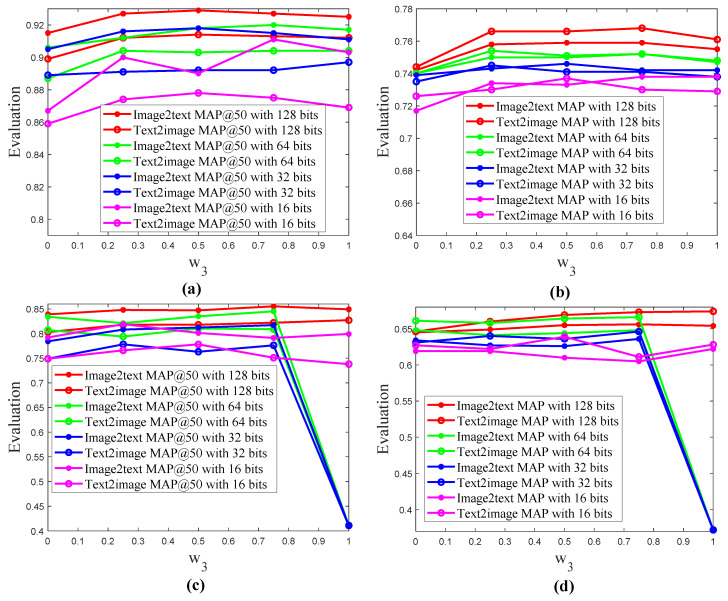
Feature space analysis on two datasets. (**a**) feature space analysis on MIRFlickr-25K with MAP@50; (**b**) feature space analysis on MIRFlickr-25K with MAP; (**c**) feature space analysis on NUS-WIDE with MAP@50; and (**d**) feature space analysis on NUS-WIDE with MAP.

During feature fusion, $w_{1}$ and $w_{2}$ were equal and both related to $w_{3}$, so we only need to display the parameter sensitivity analysis of $w_{3}$. In Figure 4, the feature space based on feature fusion has better results. Specifically, when the values of $w_{3}$, $w_{2}$ and $w_{1}$ were 0.5, 0.25 and 0.25, respectively, DSPRH retrieval performance was the best.

In Figure 4, the feature space based on feature fusion had better results. When w3 was equal to 1, w1 and w2 were both 0, which means that no feature fusion was performed. At this time, the MAP and MAP@50 evaluations of DSPRH were both unstable. When w3 was equal to 0.75 and w1 and w2 were both 0.125, DSPRH evaluation performance was relatively stable. Specifically, in Figure 4c,d as w3 increased, DSPRH obtained better performance. However, when w3 reached the maximum value, the performance of DSPRH was unstable. Therefore, when w3, w2 and w1 were 0.75, 0.125 and 0.125, respectively, DSPRH retrieval performance is the best.

In Figure 3, we show the feature space reconstruction method, and the performance analysis of the proposed feature fusion strategy is shown in Figure 4. We found that when w3 is equal to 1, the algorithm retrieval performance did not achieve the optimal value in Figure 4, so the image feature space reconstruction method of weighted fusion can obtain better retrieval performance. Therefore, the image feature space of DSPRH algorithm was implemented in Figure 3 and Figure 2 based on the proposed feature space reconstruction method. This also fully confirmed that the proposed multi-feature fusion feature extraction module was very effective in Figure 3.

#### 4.3.3. Hyper-Parameter Sensitivity Analysis

**Figure 5:**
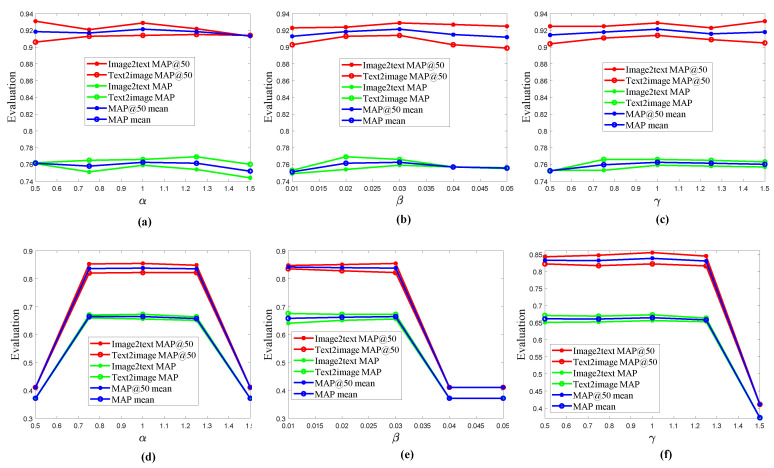
Hyper-parameter sensitivity analysis on two datasets. (**a**) parameter α analysis on MIRFlickr-25K in formula 19; (**b**) parameter β analysis on MIRFlickr-25K in formula 19; (**c**) parameter γ analysis on MIRFlickr-25K in formula 19; (**d**) parameter α analysis on NUS-WIDE in formula 19; (**e**) parameter β analysis on NUS-WIDE in formula 19; and (**f**) parameter γ analysis on NUS-WIDE in formula 19.

There are four hyper-parameter sensitivities (λ in Equation (12); α, β and γ in formula 19) which need to be determined in DSPRH. The value of the λ was 0.9, which follows the standards of DSAH [13] and DJSRH [12]. Next we analyzed the impact of the other three parameters. DSPRH chooses six indicators to discuss the validity of the parameters. Among them, Figure 5 contained 6 retrieval evaluation indicators (Image2text MAP@50, Image2text MAP, Text2image MAP@50, Text2image MAP, MAP@50 and MAP).

In Figure 5a, when the value of parameter α was 1, the DSPRH had the best performance on MIRFlickr-25K. Therefore, the parameter α of DSPRH is 1 in this paper. In Figure 5b, when the value of parameter β was 0.03, the DSPRH had the best performance on MIRFlickr-25K. Therefore, the parameter β of DSPRH is 0.03. In Figure 5c, when the value of parameter γ was 1, the DSPRH had the best performance on MIRFlickr-25K. Therefore, the parameter γ of DSPRH is 1 in this paper.

In Figure 5d, when the value of parameter α was 1, the DSPRH had the best performance on NUS-WIDE. Therefore, the parameter α of DSPRH is 1. In Figure 5e when the value of parameter β was 0.03, the DSPRH had the best performance on NUS-WIDE. Therefore, the parameter β of DSPRH is 0.03. In Figure 5f, when the value of parameter γ was 1, the DSPRH had the best performance on NUS-WIDE. Therefore, the parameter γ of DSPRH is 1.

In short, the three parameters (α, β and γ) of DSPRH have the same parameter settings on two datasets (MIRFlickr-25K and NUS-WIDE). Specifically, in Equation (19), the values of α, β and γ are 1, 0.03 and 1, respectively. Therefore, the proposed DSPRH has better parameter adaptability in cross-modal retrieval tasks.

#### 4.3.4. Other Ablation Analysis

We showed that the precision-recall rate curve and the change curve of precision with top-K are at 128 bits. DJSRH [12] and DSAH [13] were the two baselines most relevant to DSPRH, so we perform other ablation analyses (precision-recall, precision @top-K).

**Figure 6:**
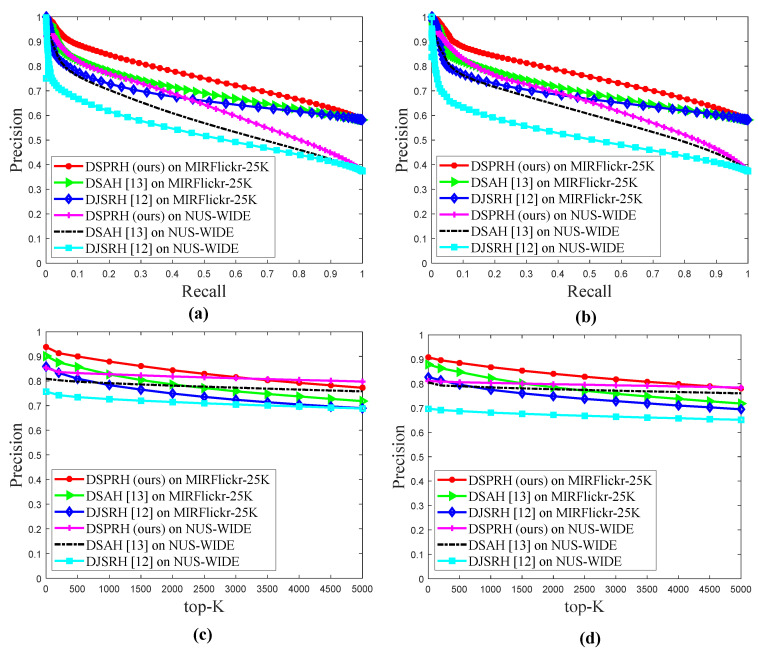
Other ablation analysis on two datasets. (**a**) Image2text precision-recall curve with 128 bits; (**b**) Text2image precision-recall curve with 128 bits; (**c**) Image2text precision @ top-K curve with 128 bits; and (**d**) Text2image precision @ top-K curve with 128 bits.

Figure 6a shows Image2text precision-recall curve. Figure 6b shows text2image precision-recall curve. Figure 6a,b shows that the DSPRH achieved better Image2text and text2image precision-recall curve performance than DJSRH [12] and DSAH [13]. Figure 6c shows Image2text precision @top-K curve. Figure 6d shows text2image precision @top-K curve. Figure 6c,d show that the DSPRH achieved better Image2text and text2image precision @top-K curve performance than DJSRH [12] and DSAH [13].

DJSRH [12] was proposed in ICCV 2019, the main goal is to reconstruct the loss function. The algorithm reconstructs the semantic loss based on image modal and text modal features. DJSRH [12] achieved better performance, but the algorithm did not consider semantic alignment loss. Compared with DJSRH, DSAH [13] considers semantic alignment loss, so image modal semantics and text modal semantic information are aligned and associated. DSAH [13] was proposed in ICMR 2020. Encouraged by the DSAH algorithm, we propose the DSPRH algorithm. We used the idea of feature fusion to reconstruct the image feature space, and propose a new cross-modal retrieval loss function based on considering multiple modal semantic alignment loss, pairwise loss and cosine triple loss.

In this way, semantic alignment loss can reduce the semantic differences between the two modalities, and the pairwise loss and cosine triple loss can shorten the distance between similar samples between the two modalities and maintain the differences between dissimilar samples between the two modalities. Figure 6 further shows that the proposed DSPRH has better retrieval performance, which also fully demonstrates that the proposed technology is scalable and suitable for cross-modal retrieval tasks. Therefore, the experimental chapter shows that the proposed algorithm has better retrieval performance, and further elaborates the reliability and effectiveness of the proposed algorithm.

## 5. Conclusions

In this work, we propose a novel unsupervised cross-modal hash retrieval method, named deep semantic-preserving reconstruction hashing. This work uses tensor regular-polymorphic decomposition theory, two bottlenecks auto-encoding and semantic-preserving reconstruction loss to complete the cross-modal retrieval task. The specific description is as follows: In the original feature space, we use tensor regular-polymorphic decomposition theory to generate rank-1 tensor to capture high-order context modal information. This can assist the backbone network to capture important modal semantic information. Based on second-order statistical theory, we use global covariance pooling to capture channel semantic information and accelerate network convergence. In order to achieve modal and semantic interaction, we propose a two bottlenecks self-encoding feature reconstruction layer to achieve visual-text modal information interaction. In the metric learning part, we design a new semantic-preserving reconstruction loss to achieve modal alignment and information reconstruction, which can reserve important modal semantic information. The experiment was performed on two datasets (MIRFlickr-25K and NUS-WIDE), and the paper gives detailed experimental evaluation, parameter setting, baseline comparison and ablation analysis. Experimental results show that DSPRH has achieved better retrieval performance.

The proposed method has good adaptability to perform cross-modal retrieval tasks, and we do not need to adjust the parameters of the loss function to deal with different cross-modal retrieval tasks. In this work, the study found that the construction of the loss function is the key to cross-modal retrieval, and the metric learning strategy is the key to affecting the performance of cross-modal retrieval. When reconstructing the feature space, the proposed algorithm adopts a multi-feature fusion strategy. At this time, the parameters need to be fine-tuned and manually adjusted. In future research, we will continue to explore key technologies for cross-modal retrieval, such as image-text retrieval, image-text matching and video-text retrieval.

## Figures and Tables

**Table 1:** Definition of basic symbols.

Symbol Definition	Symbol Interpretation
$F_{I}$	Image feature space
$F_{T}$	Text feature space
$O_{k}$	*k*-th input image-text semantic pair
$F_{I}^{\prime }$	Reconstruct image feature space
$F_{T}^{\prime }$	Restructure text feature space
$I_{k}$	*k*-th semantic image feature
*T_k_*	*k*-th semantic text feature

**Table 2:** Performance comparison on MIRFlickr-25K.

Algorithm	Journal		MIRFlickr-25K							
	Year	Backbone	Image2text				Text2image			
			16	32	64	128	16	32	64	128
MAP										
**DSPRH(ours)**	**-**	**ResNet34**	**0.762**	**0.767**	**0.775**	**0.781**	**0.758**	**0.772**	**0.783**	**0.796**
**DSPRH(ours)**	**-**	**AlexNet**	**0.733**	**0.746**	**0.752**	**0.759**	**0.737**	**0.741**	**0.752**	**0.766**
UKD-SS [14]	CVPR, 2020	VGG19	0.714	0.718	0.725	0.720	0.715	0.716	0.721	0.719
SRCH [11]	IJCAI, 2020	VGG16	0.681	0.692	0.700	-	0.697	0.708	0.715	-
MGAH [33]	TMM, 2020	VGG19	0.685	0.693	0.704	0.702	0.673	0.676	0.686	0.690
UGACH [9]	AAAI, 2018	VGG19	0.685	0.693	0.704	0.702	0.673	0.676	0.686	0.690
DJSRH [12]	ICCV, 2019	AlexNet	0.647	0.651	0.664	0.680	0.639	0.653	0.669	0.683
DSAH [13]	ICMR, 2020	AlexNet	0.684	0.690	0.699	0.707	0.687	0.703	0.699	0.709
DBRC [28]	TMM, 2019	-	0.592	0.592	0.585	0.591	0.594	0.595	0.594	0.590
CRE [22]	TIP, 2019	-	0.621	0.625	0.629	-	0.615	0.618	0.622	-
MAP@50										
**DSPRH(ours)**	**-**	**ResNet34**	**0.932**	**0.937**	**0.944**	**0.951**	**0.892**	**0.910**	**0.917**	**0.924**
**DSPRH(ours)**	**-**	**AlexNet**	**0.890**	**0.918**	**0.920**	**0.929**	**0.878**	**0.892**	**0.904**	**0.914**
DJSRH [12]	ICCV, 2019	AlexNet	0.776	0.805	0.836	0.863	0.749	0.790	0.825	0.84
DSAH [13]	ICMR, 2020	AlexNet	0.855	0.875	0.890	0.900	0.850	0.870	0.877	0.883
DADH [15]	ICMR, 2020	CNN-F	0.802	0.807	0.818	-	0.792	0.796	0.806	-
AGAH [16]	ICMR, 2019	CNN-F	0.792	0.794	0.807	-	0.789	0.790	0.805	-
UDCMH [6]	IJCAI, 2018	AlexNet	0.689	0.698	0.714	0.717	0.692	0.704	0.718	0.733
SCH-GAN [34]	TOC, 2020	VGG19	0.738	0.745	0.757	0.768	0.771	0.790	0.793	0.804
DSPOH [29]	TNNLS, 2019	VGG16	0.853	0.859	-	-	0.834	0.848	-	-
DSPOH [29]	TNNLS, 2019	AlexNet	0.832	0.840	-	-	0.832	0.841	-	-
SCRATCH [23]	TCSVT, 2019	CNN-F	0.723	0.741	0.766	0.776	0.798	0.818	0.842	0.851
MTFH [10]	TPAMI, 2019	-	0.747	0.761	0.765	0.768	0.804	0.815	0.817	0.835
EGDH [37]	IJCAI, 2019	CNN-F	0.757	0.773	0.796	0.790	0.779	0.794	0.799	0.801
DMFH [31]	TCSVT, 2020	CNN-F	0.780	0.792	0.795	-	0.798	0.810	0.810	-
BATCH [26]	TKDE, 2020	CNN-F	0.738	0.744	0.745	0.749	0.821	0.829	0.835	0.838
ATFH-N [25]	TETCI, 2020	VGG19	0.734	0.748	0.733	0.722	0.790	0.803	0.794	0.768
MDCH [32]	TMM, 2020	VGG19	0.805	0.822	0.834	-	0.806	0.817	0.823	
CPAH [27]	TIP, 2020	VGG16	0.789	0.796	0.795	-	0.778	0.786	0.785	--
MLCAH [30]	TMM, 2020	CNN-F	0.796	0.808	0.815	-	0.794	0.805	0.805	-
SSAH [5]	CVPR, 2018	VGG19	0.797	0.809	0.810	-	0.782	0.797	0.799	-
SSAH [5]	CVPR, 2018	CNN-F	0.782	0.790	0.800	-	0.791	0.795	0.803	-

**Table 3:** Performance comparison on NUS-WIDE.

Algorithm	Journal		NUS-WIDE							
	Year	Backbone	Image2text				Text2image			
			16	32	64	128	16	32	64	128
MAP										
**DSPRH(ours)**	**-**	**ResNet34**	**0.639**	**0.661**	**0.667**	**0.667**	**0.637**	**0.67**	**0.68**	**0.699**
**DSPRH(ours)**	**-**	**AlexNet**	**0.605**	**0.636**	**0.648**	**0.656**	**0.611**	**0.646**	**0.666**	**0.673**
UKD-SS [14]	CVPR, 2020	VGG19	0.614	0.637	0.638	0.645	0.630	0.656	0.657	0.663
SRCH [11]	IJCAI, 2020	VGG16	0.544	0.557	0.567	-	0.553	0.567	0.575	-
MGAH [33]	TMM, 2020	VGG19	0.613	0.623	0.628	0.631	0.603	0.614	0.640	0.641
UGACH [9]	AAAI, 2018	VGG19	0.613	0.623	0.628	0.631	0.603	0.614	0.640	0.641
DJSRH [12]	ICCV, 2019	AlexNet	0.476	0.491	0.517	0.537	0.474	0.501	0.523	0.533
DSAH [13]	ICMR, 2020	AlexNet	0.566	0.587	0.599	0.599	0.563	0.593	0.603	0.631
DBRC [28]	TMM, 2019	-	0.393	0.404	0.410	0.402	0.425	0.421	0.428	0.436
CRE [22]	TIP, 2019	-	0.513	0.530	0.533	0.534	0.493	0.509	0.515	0.516
MAP@50										
**DSPRH(ours)**	**-**	**ResNet34**	**0.817**	**0.848**	**0.851**	**0.863**	**0.774**	**0.805**	**0.820**	**0.842**
**DSPRH(ours)**	**-**	**AlexNet**	**0.791**	**0.817**	**0.845**	**0.855**	**0.751**	**0.776**	**0.809**	**0.822**
DJSRH [12]	ICCV, 2019	AlexNet	0.663	0.688	0.741	0.772	0.626	0.677	0.703	0.715
DSAH [13]	ICMR, 2020	AlexNet	0.772	0.789	0.811	0.826	0.741	0.777	0.787	0.811
DADH [15]	ICMR, 2020	CNN-F	0.649	0.666	0.666	-	0.650	0.668	0.681	-
AGAH [16]	ICMR, 2019	CNN-F	0.646	0.660	0.651	-	0.631	0.642	0.634	-
UDCMH [6]	IJCAI, 2018	AlexNet	0.511	0.519	0.524	0.558	0.637	0.653	0.695	0.716
SCH-GAN [34]	TOC, 2020	VGG19	0.713	0.724	0.732	0.749	0.738	0.742	0.769	0.782
DSPOH [29]	TNNLS, 2019	VGG16	0.701	0.723	-	-	0.737	0.753	-	-
DSPOH [29]	TNNLS, 2019	AlexNet	0.695	0.711	-	-	0.713	0.731	-	-
SCRATCH [23]	TCSVT, 2019	CNN-F	0.643	0.649	0.67	0.673	0.789	0.807	0.827	0.832
MTFH [10]	TPAMI, 2019	-	0.655	0.659	0.676	0.675	0.757	0.780	0.795	0.804
EGDH [37]	IJCAI, 2019	CNN-F	-	--	-	-	-	-	-	-
DMFH [31]	TCSVT, 2020	CNN-F	0.631	0.647	0.680	-	0.607	0.621	0.640	-
BATCH [26]	TKDE, 2020	CNN-F	0.627	0.651	0.669	0.669	0.760	0.778	0.782	0.784
ATFH-N [25]	TETCI, 2020	VGG19	0.615	0.611	0.620	0.605	0.695	0.714	0.722	0.699
MDCH [32]	TMM, 2020	VGG19	0.665	0.682	0.692	-	0.692	0.699	0.707	-
CPAH [27]	TIP, 2020	VGG16	0.607	0.627	0.634	-	0.642	0.662	0.665	-
MLCAH [30]	TMM, 2020	CNN-F	0.644	0.641	0.643	-	0.662	0.673	0.687	-
SSAH [5]	CVPR, 2018	VGG19	0.636	0.636	0.637	-	0.653	0.676	0.683	-
SSAH [5]	CVPR, 2018	CNN-F	0.642	0.636	0.639	-	0.669	0.662	0.666	-

**Table 4:** Loss function ablation analysis on two datasets.

Bits	Evaluation	MIRFlickr-25K				NUS-WIDE			
		$L_{All}$	$L_{1}$	$L_{2}$	$L_{3}$	$L_{All}$	$L_{1}$	$L_{2}$	$L_{3}$
16	Image2test MAP@50	0.890	0.853	0.720	0.868	0.791	0.762	0.740	0.774
	Text2image MAP@50	0.878	0.858	0.648	0.853	0.751	0.761	0.713	0.772
	Image2test MAP	0.733	0.680	0.625	0.693	0.605	0.558	0.578	0.579
	Text2image MAP	0.737	0.684	0.607	0.694	0.611	0.573	0.581	0.600
32	Image2test MAP@50	0.918	0.876	0.859	0.883	0.817	0.783	0.576	0.800
	Text2image MAP@50	0.892	0.873	0.852	0.876	0.776	0.779	0.495	0.783
	Image2test MAP	0.746	0.688	0.707	0.706	0.636	0.571	0.479	0.598
	Text2image MAP	0.741	0.697	0.712	0.707	0.646	0.591	0.460	0.607
64	Image2test MAP@50	0.920	0.889	0.911	0.906	0.845	0.811	0.731	0.822
	Text2image MAP@50	0.904	0.879	0.893	0.889	0.809	0.787	0.693	0.793
	Image2test MAP	0.752	0.696	0.740	0.715	0.648	0.597	0.592	0.615
	Text2image MAP	0.752	0.697	0.743	0.715	0.666	0.602	0.594	0.622
128	Image2test MAP@50	0.929	0.901	0.926	0.911	0.855	0.826	0.833	0.835
	Text2image MAP@50	0.914	0.884	0.904	0.895	0.822	0.809	0.791	0.815
	Image2test MAP	0.759	0.707	0.748	0.725	0.656	0.598	0.636	0.615
	Text2image MAP	0.766	0.708	0.748	0.727	0.673	0.630	0.651	0.640
